# Supplementary material for: TORCH Congenital Syndrome Infections in Central America’s Northern Triangle
Source: Microorganisms. 2023 Jan 19;11(2):257. doi: 10.3390/microorganisms11020257 (PMC9964893; doi:10.3390/microorganisms11020257)
Supplement: Supplementary file 1 [file microorganisms-11-00257-s001.zip › microorganisms-2029652-supplementary.pdf]

Pathogen	Location	Population	Test Performed	Results	Article/Data Type [Reference]
T. gondii	n/a	Pregnant women at global, WHO region, country level	Secondary data analysis of serology studies, Bayesian modelling	Country level seroprevalence:<br>- Guatemala<br>IgM 0.9% (range: 0.8%-1.0%)<br>IgG 52.8% (range: 52.5%-53.2%)<br>- Honduras<br>IgM 1.0% (range: 1.0%-1.1%)<br>IgG 29.7% (range: 29.4%-30.0%)<br>- El Salvador<br>IgM 0.9% (range: 0.8%-1.0%)<br>IgG 52.1% (range: 51.7%-52.4%)	Systematic review, meta-analysis, modelling analysis [31]
T. gondii	San Juan Sacatepéquez, Guatemala	60 women presenting to “Santa Bárbara” clinic	IgM CLIA	0% (0/60) IgM positivity of women had positive IgM result	Cross-sectional analysis, Veterinary medicine thesis [36]
T. gondii	Zacapa department, Guatemala	236 woman, ages 15-45 years who previously attended an information session	IgG ELISA	50.4% (119/236) positive	Cross-sectional analysis, Biochemistry thesis [37]
T. gondii	Zacapa and Sacatepéquez departments, Guatemala	523 neonates from departmental referent hospitals Antigua Guatemala, Amatitlán y Zacapa	IgM and IgG ELISA	0.2% (1/523) IgM positive neonates<br>-positive neonate resulted from Hospital Nacional de Amatitlán confirmed with positive IgM in the mother	Cross-sectional analysis, Biochemistry thesis [38]
T. gondii	Paraíso city, Honduras	88 pregnant women presenting to prenatal care unit	IgM and IgG ELISA	48% (42/88) of women IgM positive<br>49% (43/88) of women IgG positive	Cross-sectional analysis, Epidemiology thesis [39]
T. gondii	Concepción municipality, Usulatán, El Salvador	98 women, ages 15-45 years presenting for external consult in a public health clinic	IgM and IgG combination immunochromatographic lateral flow test	0% (0/98) of women IgM positive<br>26.5% (26/98) of women IgG positive	Cross-sectional analysis, Clinical Laboratory Specialist thesis [39]
T. gondii	San Miguel, El Salvador	86 adult female blood donors care in a departmental referent public hospital	IgM and IgG ELISA	0% (0/86) of women IgM positive<br>17% (15/86) of women IgG positive	Cross-sectional analysis, Clinical Laboratory Specialist thesis [41]
T. gondii, CMV, ZIKV	San Salvador, El Salvador	Infants ages <1 year with microcephaly attending neurological consult at the national children’s hospital	Clinical chart abstraction	7.3% (3/41) infants positive for *T. gondii*<br>7.3% (3/41) infants positive for *T. gondii* and CMV<br>12.2% (5/41) infants positive for CMV<br>7.3% (3/41) infants positive for ZIKV	Cross-sectional analysis, Pediatric Medicine thesis [42]
T. cruzi	Central America	Country level	Data derived from literature, Institutes/Ministries of Health, public health official interviews	Country level seroprevalence:<br>- Guatemala: 1.2%<br>- Honduras:0.9%<br>- El Salvador: 1.3%	Review [57]
T. cruzi	Güisiltepeque y Los Riscos municipalities, Jalapa, Guatemala	150 women ages 15-49 years	IgG ELISA	3.3% (5/150) IgG positive<br>-2/5 pregnant women included in the study were IgG positive	Cross-sectional analysis, Biochemistry thesis [58]
T. cruzi	Comapa municipality, Jutiapa, Guatemala	390 women of childbearing age	IgG ELISA	10.8% (42/390) IgG positive	Cross-sectional analysis [59]
T. cruzi	Las Palmas town, Olopa, Chiquimula, Guatmala	134 women ages 15-49 years	IgG IHA, ELISA, immunochromatographic lateral flow test and microscopy	27.6% (37/134) IgG positive	Cross-sectional analysis, Biochemistry thesis [60]
T. cruzi	12 departments Honduras	2794 pregnant women and girls ages 13-39	IgG ELISA	1.6% (46/2794) IgG positive	Cross-sectional analysis, Epidemiology thesis [61]
T. cruzi	Argentina, Mexico and Honduras	6853 women presenting for delivery in two hospitals and respective neonates from La Esperanza and Santa Bárbara, Honduras	immunochromatographic lateral flow, IgG ELISA and PCR on maternal blood	-Honduras:<br>2.8% (183/6,583) cord blood samples positive by rapid test<br><br>78.7% (181/230) IgG ELISA positive mothers	Cross-sectional analysis [56]

		(28145 total women recruited from all country study sites)		72.9% (132/181) PCR positive mothers<br><br>0/182 congenital transmission cases	
T. cruzi	Honduras	Clinical suspicion of neonate based on maternal history and neonatal exam	Direct parasitological examination	-Asymptomatic at the time of birth with remarkable cardiac results on echocardiogram<br>-Infant treated with Nifurtimox and had negative blood smear at 6 months post treatment	Case Report [62]
T. cruzi	Ahuachapán & Sonsonate departments, El Salvador	943 pregnant women recruited from health centers from 4 selected high-risk municipalities	IgG ELISA, IHA	3.8% (36/943) IgG positive mothers<br><br>1 of 32 infants screened between 9-16 months positive from Guymango	Cross-sectional analysis [54]
T. cruzi	San Miguel, Morazán, La Unión departments, El Salvador	116 pregnant women and girls 10-49 years presenting for prenatal care at health centers from three towns	Strout, IgG ELISA	8.6% (10/116) participants ELISA & Strout positive<br><br>-10% (1/10) positive participants reported history of Romaña sign	Cross-sectional analysis, Medical degree thesis [63]
T. cruzi	La Unión, El Salvador	145 third trimester pregnant women and girls ages 13-41 and resulting neonates	Immunochromatographic lateral flow, IgG ELISA, Strout	1.9% (1/52) positive by ELISA and rapid test<br><br>-resulting neonate from the positive mother was negative by Strout	Cross-sectional analysis, Clinical Laboratory Specialist thesis [64]
ZIKV	WHO Region of the Americas	Reported cases at the country level 2015-2018	n/a	- Guatemala<br>3,907 reported autochthonous cases<br>1,302 confirmed cases<br>140 congenital cases<br>- Honduras<br>32,385 reported autochthonous cases<br>308 confirmed cases<br>8 congenital cases<br>- El Salvador<br>11,789 reported autochthonous cases<br>51 confirmed cases<br>4 congenital cases	PAHO data [75]
CMV	n/a	General population, blood and organ donors, and women of childbearing age at WHO region, country level	Secondary data analysis of serology studies	- El Salvador<br>87% (95%UI: 67-97)	Systematic Review, meta-analysis [95]

CLIA- chemiluminescent immunoassay, ELISA- enzyme-linked immunosorbent assay; IHA- Indirect hemagglutination CMV/ cytomegalovirus, ZIKV/ zika virus, UI- uncertainty interval, PAHO/-Pan American Health Organization, WHO- World Health Organization. Professional degree theses have not been peer-reviewed.
